# The genomic architecture of antimalarial drug resistance

**DOI:** 10.1093/bfgp/elz008

**Published:** 2019-05-23

**Authors:** Annie N Cowell, Elizabeth A Winzeler

**Affiliations:** 1 Division of Infectious Diseases and Global Health, Department of Medicine, University of California, San Diego, Gilman Dr., La Jolla, CA, USA; 2 Division of Host-Microbe Systems & Therapeutics, Department of Pediatrics, University of California, San Diego, Gilman Dr., La Jolla, CA, USA

**Keywords:** malaria, drug resistance, *Plasmodium falciparum*, *Plasmodium vivax*, artemisinin

## Abstract

*Plasmodium falciparum* and *Plasmodium vivax*, the two protozoan parasite species that cause the majority of cases of human malaria, have developed resistance to nearly all known antimalarials. The ability of malaria parasites to develop resistance is primarily due to the high numbers of parasites in the infected person’s bloodstream during the asexual blood stage of infection in conjunction with the mutability of their genomes. Identifying the genetic mutations that mediate antimalarial resistance has deepened our understanding of how the parasites evade our treatments and reveals molecular markers that can be used to track the emergence of resistance in clinical samples. In this review, we examine known genetic mutations that lead to resistance to the major classes of antimalarial medications: the 4-aminoquinolines (chloroquine, amodiaquine and piperaquine), antifolate drugs, aryl amino-alcohols (quinine, lumefantrine and mefloquine), artemisinin compounds, antibiotics (clindamycin and doxycycline) and a napthoquinone (atovaquone). We discuss how the evolution of antimalarial resistance informs strategies to design the next generation of antimalarial therapies.

## Article

Malaria, a protozoan infection caused by *Plasmodium* parasites, remains a major cause of morbidity and mortality worldwide primarily among children less than 5 years old. It caused an estimated 219 million cases and 435 000 deaths in 2017, with 92% of cases and 93% of deaths in Africa (2017 #885; 2018 #1580). *Plasmodium falciparum* and *Plasmodium vivax*, the two species that cause the majority of cases of human malaria, have demonstrated resistance to nearly all known antimalarials, with the highest levels of resistance found in *P. falciparum* in Southeast Asia. When parasite resistance to chloroquine (CQ) and antifolate medications, former first line medications, emerged, there were enormous increases in morbidity and mortality [1]. More recently, delayed parasite clearance times following artemisinin combination therapy (ACT), the current first line treatment for uncomplicated *P. falciparum* infections, have been reported in the Greater Mekong sub-region and represent a major threat to the ability to control and treat malaria [2, 3].

**Figure 1:**
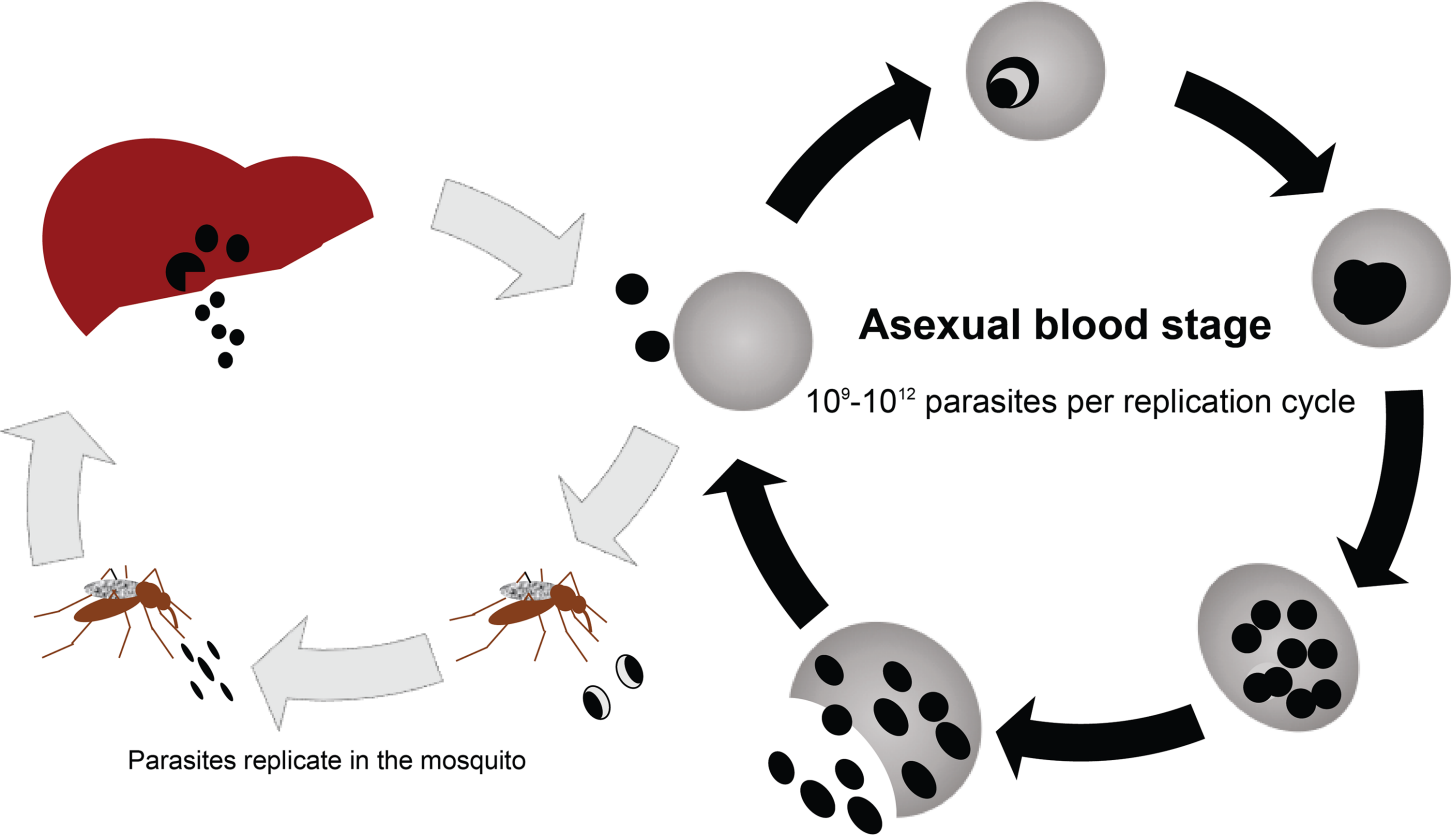
The *P. falciparum* life cycle highlighting the asexual blood stage of infection where antimalarial resistance mutations arise. Infection begins with inoculation of sporozoites by an infected mosquito. Sporozoites infect liver cells, and merozoites are released into the bloodstream, which invade red blood cells (RBCs). During the asexual blood stage of infection, which is responsible for the clinical manifestations of disease, the parasites undergo maturation and replication with an average of 10^9^–10^12^ parasites per replication cycle. The infected RBCs rupture, releasing new merozoites into the bloodstream to begin another cycle of replication. A subset of parasites becomes gametocytes which can be ingested by another mosquito to continue malaria transmission.

The ability of malaria parasites to develop resistance is primarily due to the high burden of parasites in an infected person’s bloodstream during the asexual blood stage of infection in conjunction with the mutability of the parasites’ genomes [4]. Identifying the genetic mutations that mediate antimalarial resistance is key to understanding how the parasites evade our treatments. Tracking these molecular markers in clinical samples can help evaluate the emergence of resistance in a particular region and inform recommendations for first line therapy. This is especially useful since empirical testing for drug resistance, either in patients or after taking parasites into short-term culture, can be very expensive and requires resources that are not available in many malaria-endemic regions. Our understanding of the mechanisms of antimalarial resistance is primarily focused on *P. falciparum*, for which there is a robust *in vitro* culture system. Major mechanisms of resistance include point mutations in or amplification of genes encoding transporters that mediate transport of a drug to or from the parasite’s digestive vacuole (DV) and point mutations in the target of the antimalarial that disrupt binding. Whole genome scans of *P. falciparum* and *P. vivax* using technologies such as microarrays and whole genome sequencing (WGS) have revealed insights into mechanisms of resistance in both *in vitro* and clinical studies. Genome-wide association studies (GWASs) have helped identify genes associated with resistance. In this review, we examine the genetic mechanisms that underlie resistance to the major classes of antimalarial medications and discuss how this knowledge has contributed to our understanding of developing more effective, ‘irresistible’ malaria treatments.

## The emergence and spread of antimalarial resistance

Resistance is defined as the ability of a parasite to survive or multiply despite properly administered and dosed medication [5]. Currently, antimalarials are administered as combination therapy with two drugs to prevent the rapid emergence of resistance. As levels of resistance increase, there is an increased number of patients presenting with late recrudescence, or persistent parasitemia [4]. In addition, patients present with recrudescence earlier following treatment. High-grade resistance is evident when there is failure to clear parasitemia or there is an increase in parasitemia despite appropriate therapy. An important marker of resistance is delayed parasite clearance times. A major challenge with assessing antimalarial efficacy in the era of combination therapy is that failure may not be observed even when the parasites are resistant to one of the partner drugs.

The first step in the development of resistance is the initial genetic event, which is thought to be spontaneous and rare [4]. Since an average human infection can comprise 10^9^–10^13^ parasites in the blood stream during the asexual blood stage (Figure 1) with an estimated 1.0–9.7 × 10^−9^ mutations per base pair per generation [6], there is a high likelihood that a random mutation can occur that leads to antimalarial resistance within a few cycles of replication. Subsequent selection for that mutation occurs due to a survival advantage in the presence of drug pressure. Factors that favor selection of resistant parasites are higher levels of parasitemia, decreased blood levels of antimalarials and decreased patient immunity [4, 7]. Drugs with a longer drug half-life such as mefloquine (MFQ), piperaquine (PPQ) and CQ may be more likely to select for resistance [8]. The level of malaria transmission also can affect the development of resistance since persons in low transmission settings are more likely to be symptomatic and receive treatment compared to those in high transmission settings [4]. Individuals in lower transmission areas also have lower acquired immunity, which can result in increased transmission of resistant parasites. In high transmission settings, there are more likely to be multiple genotypes present in a single infection and thus resistant parasites have to compete with wild-type parasites. In areas with seasonal malaria transmission, however, persons with asymptomatic parasitemia can serve as a reservoir for sensitive parasites [9]. The transmissibility of the allele is also an important consideration and may determine whether resistance can spread from patient to patient. For example, some alleles that confer resistance to atovaquone cause parasites to die in the mosquitos so that they should, in principle, not spread from one person to the next [10].

## Known genetic mediators of resistance

### 4-Aminoquinolines

**Figure 2:**
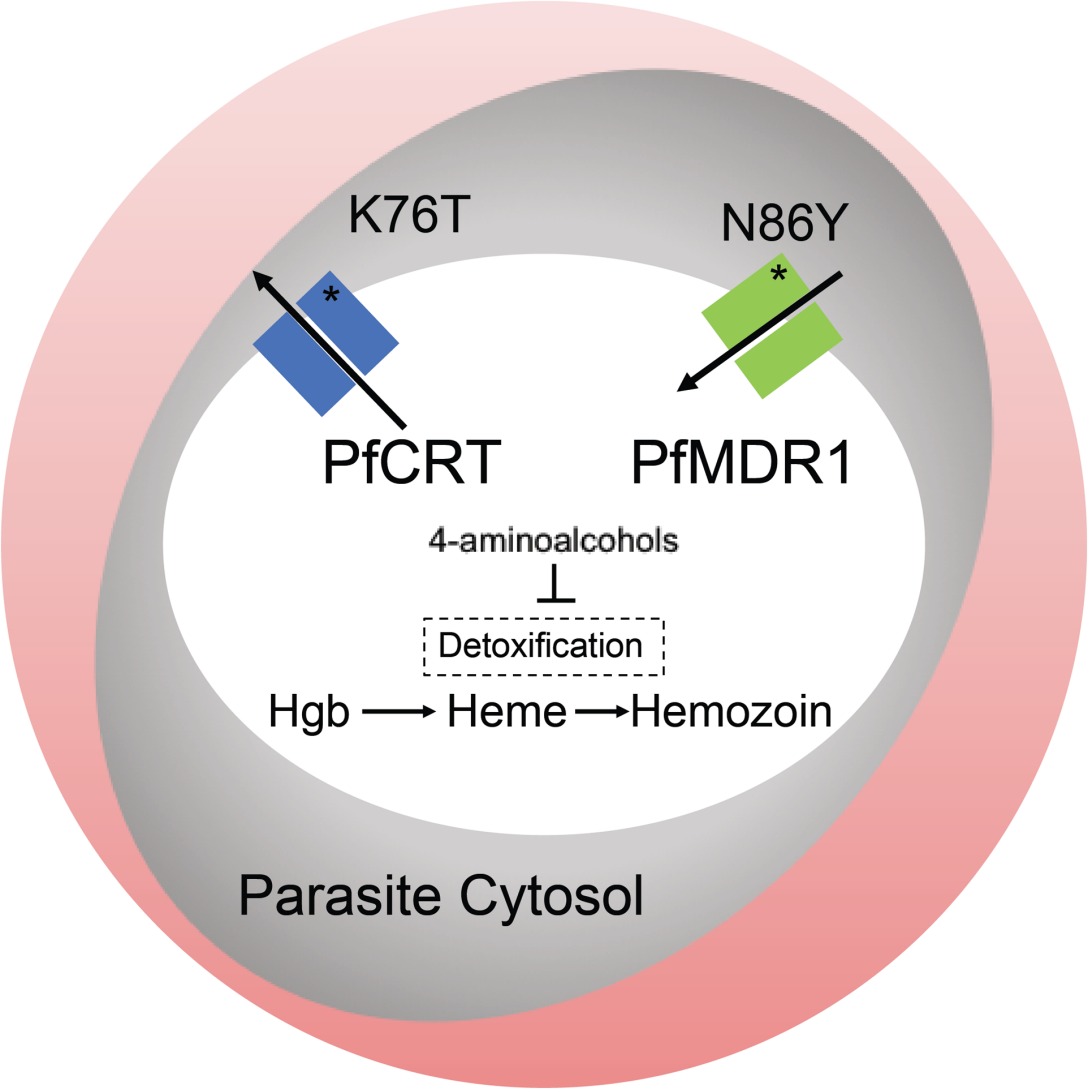
The parasite DV and the role of the *P. falciparum* CQ resistance transporter (PfCRT) and the *P. falciparum* multidrug resistance protein 1 (PfMDR1). The parasite (gray oval) is shown within an RBC. The DV (white oval) is a compartment within the parasite where the catabolism of hemoglobin (Hgb) from the host RBC occurs. The breakdown of Hgb results in reactive heme which undergoes detoxification to hemozoin. Medications from the 4-aminoquinoline class bind heme and interfere with detoxification. PfCRT and PfMDR1 are DV membrane proteins. It is thought that PfCRT transports drugs out of the DV while PfMDR1 transports them into the DV [19, 36]. The T mutation in *pfcrt* is essential to CQ resistance, while the N86Y mutations in *pfmdr1* augment CQ resistance. Mutations in these transporters have also been found to mediate resistance to the aryl-amino alcohols and artemisinin.

The 4-aminoquinolines include CQ, amodiaquine (AQ) and PPQ (Table 1). CQ was previously the first-line treatment for uncomplicated *P. falciparum* infections, while AQ and PPQ are currently used as partner drugs for artemisinin derivatives. Hemoglobin catabolism in the DV of the parasite is important as a source of amino acids (Figure 2). The breakdown of hemoglobin releases Fe^2+^ iron-containing reactive heme moieties that undergo oxidation in the DV into ferriprotoporphyrin IX (FPIX) [11]. This process causes oxidative stress, and thus FPIX undergoes detoxification by becoming incorporated into hemozoin [12]. Medications from this class bind to the reactive heme and interfere with its detoxification. CQ is a weak base at a neutral pH that can diffuse across membranes into the erythrocyte and DV in its uncharged form. Once it is in the acidic DV, becomes protonated and accumulates in the DV [13, 14].

**Table 1:** Commonly used antimalarials and their known genetic mediators of resistance in *P. falciparum* and *P. vivax*. SNVs known to be essential to resistance are highlighted with an asterisk

**Antimalarial drug class**	**Mechanism of action**	**Specific drugs**	**Genetic mediator(s) of resistance**	
			***P. falciparum***	***P. vivax***
4-aminoquinolines	Interfere with heme detoxification	chloroquine (CQ)	SNVs in *pfcrt* (K76 T*); SNVs in *pfmdr1* (N86Y*)	Not well understood; *pvcrt-o* amplification
		amodiaquine(AQ)		
		piperaquine (PPQ)	SNVs in *pfcrt* (C101F, H97Y, F145I, M343 L, G353 V); Plasmepsin 2 and 3 amplifications; *pfmdr1* single copy	
4-aminoquinolines	Unknown	Primaquine	Unknown	Unknown
		Tafenaquine		
Antifolate drugs	Inhibition of folate synthesis	DHFR inhibitors (proguanil, pyrimethamine)	SNVs in *pfdhfr* (S108 N, N51I, C59R, I164L); amplification of *gtp* cyclohydrolase 1	SNVs in *pvdhfr*
		Sulfa drugs (sulfamethoxazole, sulfadoxine)	SNVs in SNVs in *pfdhps*	Inherently resistant due to SNV in *pvdhps* (V585)
Aryl amino-alcohols	Unclear; thought to interfere with heme detoxification	lumefantrine (LMF)	Amplification of *pfmdr1*	Amplification of *pvmdr1*
		mefloquine (MFQ)		
		Quinine	Not clear, involves mediators of LMF and MQ resistance; ms4760 microsatellites in *pfnhe-1*	Not reported
Antibiotics	Inhibition of protein synthesis	Doxycycline	Unknown	Not reported
		Clindamycin	SNV in 23S rRNA (A1875C)	
Napthoquinones	Inhibits cytochrome bc_1_ complex	Atovaquone	SNV in *cyt-b* (Y268S/C/N)	Not reported
Artemisinin compounds	Causes oxidative stress	Artemisinin, artemether, DHA	SNVs in *kelch13* (C580Y)	Not reported

CQ was the most widely used antimalarial in this class prior to the development of widespread resistance in *P. falciparum* and *P. vivax* in certain areas. It was introduced in the 1950s and 1960s and was used as the basis of the World Health Organization (WHO) Global Malaria Eradication Program. *P. falciparum* resistance subsequently appeared in Southeast Asia in the late 1950s, then emerged in other countries in Asia, South America and finally Africa over the course of 30 years [15, 16]. Resistance is primarily determined by mutations in *pfcrt*, a gene that encodes the CQ resistance transporter (PfCRT), a 424 amino acid protein that localizes to the DV membrane [17–19]. This is a highly polymorphic protein with over 20 different point mutations described [20, 21]. However, the K76T mutation was found to be essential for *in vitro* CQ resistance [17, 19, 22] (Table 1). Reversal of the K76T mutation resulted in wild-type CQ susceptibility and led to increased binding of CQ to FPIX [23]. Parasites harboring the K76T mutation demonstrate an increased leak of H+ from the DV in the presence of CQ compared to sensitive parasites [24]. The loss of the positively charged lysine 76 leads to increased efflux of protonated CQ from the DV [24, 25]. One study which expressed wild-type and mutant PfCRT on *Xenopus laevis* oocytes elegantly demonstrated that CQ resistance is due to direct transport of a protonated form of CQ through the K76T *pfcrt* mutant [19]. In CQR-resistant parasites that have arisen independently around the world, there are at least 4 and up to 10 additional mutations in *pfcrt* that are seen [26]. A follow-up study using the *Xenopus* system to express over 100 variants of PfCRT found that although there were many mutational routes that could confer CQ transport, the overall process was rigid, requiring that mutations were in a specific order [27]. Clinical studies have demonstrated that there was a selective sweep at the *pfcrt* locus worldwide due to CQ pressure [28–33]. The K76T mutation was also found to be associated with clinical failures [34].

The *pfmdr1* gene encodes the p-glycoprotein transmembrane pump multidrug resistance protein 1, which localizes to the DV membrane [35]. This pump functions as a transporter of antimalarial drugs, with studies demonstrating that it imports solutes into the DV [36, 37]. An N86Y mutation has been associated with decreased CQ susceptibility *in vitro* [38, 39]; however mutations in *pfmdr1* alone are not adequate to cause CQ resistance [28, 40]. Introduction of the *pfmdr1* N86Y mutation into parasites with a CQ-resistant genetic background leads to increased resistance to CQ and monodesethylamodiaquine (md-ADQ; the primary metabolite of AQ) [41].

Interestingly, after CQ was no longer used as therapy for *P. falciparum* due to widespread resistance, the K76T mutation reverted to the wild-type *pfcrt* allele in parts of Africa [42–45], suggesting that the mutation confers a loss of fitness. However, in Southeast Asia and South America this has not been the case, with the mutation persisting [46–48]. One likely reason for this is the continued use of CQ for treatment of *P. vivax* in these regions. Another potential reason is that resistance-conferring mutations are fixed in certain populations and thus there are no sensitive parasites to emerge following withdrawal of drug pressure. However, widespread CQ pressure has led to many variants of PfCRT throughout the world. In one study, researchers genetically engineered several sets of *pfcrt* mutations found at different sites around the world into *P. falciparum* parasites. They found that each PfCRT variant conferred varying degrees of CQ resistance and affected growth *in vitro*. One highly mutated *pfcrt* variant of Cambodian origin actually demonstrated enhanced growth compared to wild-type parasites [49]. Interestingly, a GWAS with CQ sensitive and resistant isolates in French Guiana found that a C350R PfCRT variant was associated with the restoration of CQ susceptibility [50]. This C350R variant was also associated with PPQ resistance *in vitro*, which likely explains the failure of PPQ in the region.

In contrast to *P. falciparum*, CQ resistance in *P. vivax* was not reported until 1989 in Papua New Guinea [51] and is now found throughout Southeast Asia and some countries in South America [52]. CQ resistance is more challenging to detect with this species since parasitemia is generally lower relative to *P. falciparum*. Additionally, it is difficult to distinguish recrudescence (parasites returning after incomplete or ineffective treatment) from relapses due to reactivation of dormant liver parasites in endemic settings. There is also no robust *in vitro* culture system as there is with *P. falciparum*, so confirmation with *in vitro* susceptibility testing is even more challenging than with *P. falciparum*. There are no clear molecular markers of CQ resistance in *P. vivax*. Although *pvcrt-o* is orthologous to *pfcrt*, there is no clear direct association between CQ resistance and mutations in *pvcrt-o* [53–55]. There is also no clear association between *pvmdr1*, the homologue of *pfmdr1* and CQ resistance. Although some studies have detected point mutations in *pvmdr1* in resistant parasite populations, such as a Y976F substitution in Indonesia and an F1076L mutation in Southeast Asia [54], the polymorphisms are not consistent across different parasite populations. In addition, there are CQ-resistant parasites that have the wild-type *pvmdr1* gene [56]. One recent study of patients with recurrent *P. vivax* infections in the Brazilian Amazon found that CQ resistance was associated with increased copies of *pvcrt-o* [57].

There is currently evidence of PPQ resistance in Western Cambodia, where dihydroartemisinin–piperaquine has been the frontline treatment for uncomplicated *P. falciparum* malaria [58]. A GWAS study of 297 *P. falciparum* clinical isolates from Cambodia found that a nonsynonymous SNP on chromosome 13, a single copy of *pfmdr1* and amplifications of *plasmepsin 2* and *3* were associated with increased *in vitro* PPQ resistance and decreased clinical efficacy [59]. Another study of culture-adapted parasites from clinical isolates from Cambodia found that *ex vivo* PPQ survival assay profiles correlated with *plasmepsin 2* copy number [60]. In addition, multicopy *plasmepsin 2* was significantly associated with DHA-PPQ treatment failure. The *plasmepsin* genes encode aspartic proteases that function as hemoglobinases in the DV. The mechanism of resistance is not clearly identified; however one hypothesis is that increased hemoglobin digestion due to the amplification decreases concentrations of the reactive heme species that PPQ binds, thereby overcoming the inhibition of heme detoxification by PPQ [60].

There is also growing evidence that mutations in *pfcrt* can mediate resistance to PPQ independent from amplifications of *plasmepsin* genes. PPQ-resistant strains evolved *in vitro* were analyzed with microarrays and were found to have a C101F mutation in *pfcrt* in addition to an amplification of *pfmdr1* [61]. Subsequently, the introduction of the C101F *pfcrt* mutation with zinc finger nuclease-based gene editing into CQ-resistant parasites resulted in significantly higher PPQ resistance and also reversed CQ resistance [62]. Three independent *pfcrt* mutations were associated with *ex vivo* PPQ resistance in culture-adapted parasites from Cambodia [63]. A GWAS study of samples primarily from Cambodia identified a point mutation in *pfcrt* (F145I), which was associated with DHA-PPQ treatment failure even after adjustment for amplification in *plasmepsin 2* and *3* [64]. A subsequent analysis of *pfcrt* allelic diversity from clinical isolates from Southeast Asia found a rapid rise in novel mutations following the introduction of DHA-PPQ treatment [65]. Introduction of the H97Y, F145I, M343L and G353V mutations into PPQ sensitive parasites resulted in PPQ resistance.

### 8-Aminoquinolines

The 8-aminoquinolines have a similar structure to the 4-aminoquinolines, with the exception of the amino group at the 8-position of the quinoline. Their mechanism of action is not well understood. Primaquine and tafenoquine are two agents that are used for malaria treatment and prophylaxis. Primaquine is given along with CQ to treat the liver-stage parasites in *P. vivax* and *P. ovale* infections to prevent relapses [66]. It also has potent activity against stage V gametocytes of *P. falciparum* and is used to reduce malaria transmission [67]. Tafenoquine was recently FDA-approved for the prevention of *P. vivax* relapses administered as a single dose. Interestingly, primaquine appears to increase the activity of CQ against CQ-resistant *P. falciparum* [68]. Primaquine resistance in *P. vivax* is difficult to determine as it is confounded by reinfections in malaria-endemic regions [69]. A study that performed WGS of *P. vivax* from known relapses that occurred despite primaquine treatment found polymorphisms in several putative resistance genes [70]. However, there are currently no known genetic markers of primaquine resistance.

### Antifolate drugs

**Figure 3:**
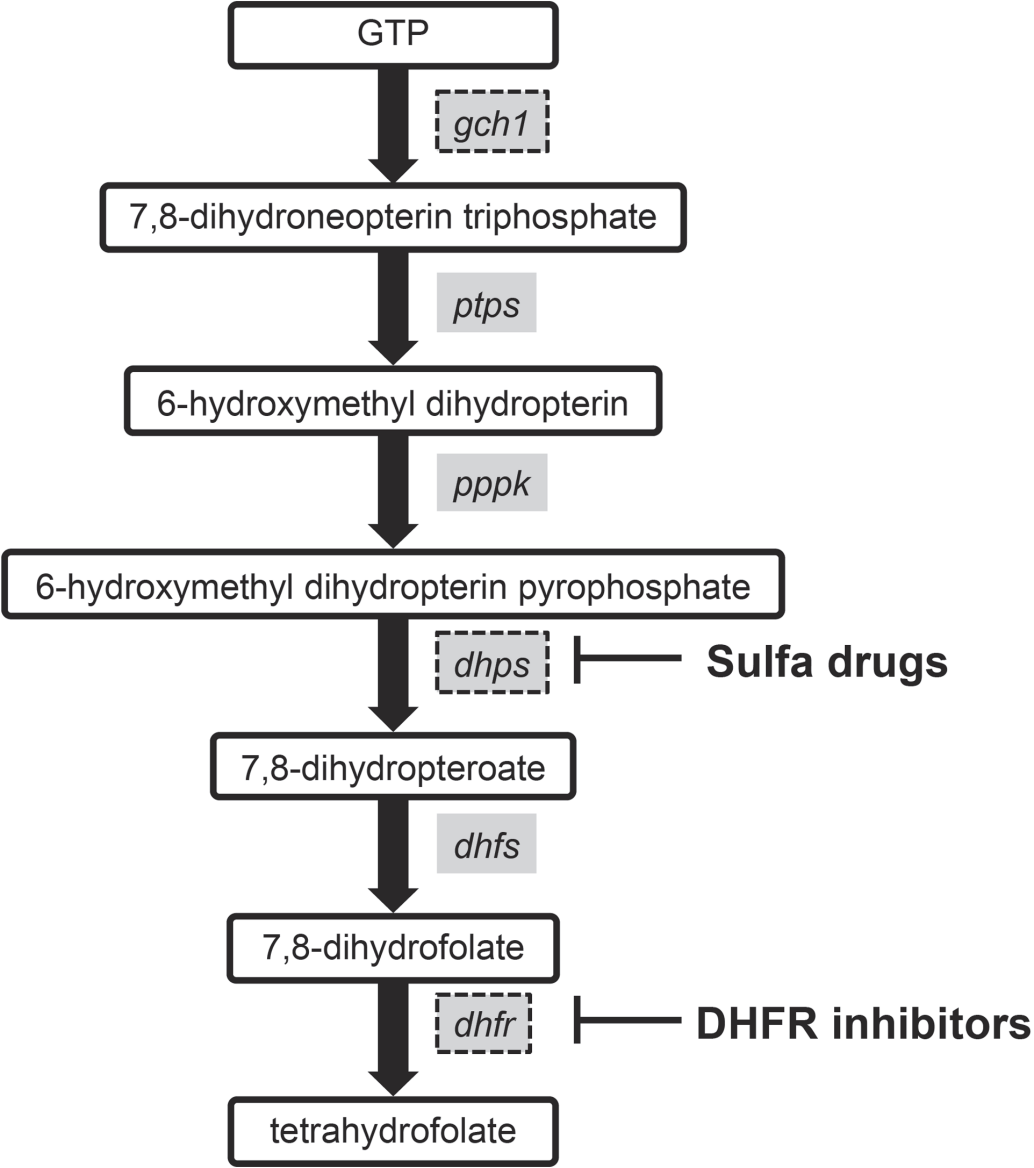
The *P. falciparum* folate biosynthesis pathway. Enzymes inhibited by the antifolate drugs are shown. Point mutations in the *dhps* and *dhfr* mediate resistance to sulfa drugs and DHFR inhibitors, respectively. Increased copy number of the *gch1* gene has been detected in clinical isolates from Southeast Asia and likely represents an adaptive evolutionary response to antifolate pressure [95]. Other abbreviations: pyruvoyltetrahydropterin synthase (*ptps*), hydroxymethyldihydropterin pyrophospholkinase (*pppk*) and dihydrofolate synthase (*dhfs*). Adapted from [94].

Antifolate drugs disrupt parasite folate synthesis (Figure 3) and include dihydrofolate reductase (DHFR) inhibitors (proguanil, pyrimethamine, trimethoprim) and sulfa drugs (sulfamethoxazole, sulfadoxine; Table 1). Sulfadoxine–pyrimethamine (Fansidar; SP) was deployed in the 1960s in areas where *P. falciparum* CQ resistance had developed, with the emergence of resistant parasites in the 1970s and 1980s [71]. Antifolates are now used most commonly as combination therapy such as atovaquone-proguanil, which is used for prophylaxis, and SP which is used in combination with artemisinin for treatment of *P. falciparum* or as part of intermittent preventive treatment in pregnant women and children.

In contrast with CQ resistance, which took many years to develop, resistance to antifolates developed much faster. The genetic mechanism of resistance to antifolates is more straightforward in comparison to CQ resistance, with single point mutations in the genes encoding either DHFR or dihydropteroate synthase (DHPS) in response to sulfa drugs (Table 1; Figure 3). These mutations cause resistance by altering binding of the drug to the enzyme’s active site [72, 73]. Three studies of microsatellites flanking the *pfdhfr* gene in *P. falciparum* clinical isolates from Thailand, South America and Southeast Africa found that in each of the locations there was a common origin of emergence [74–77]. The *pfdhfr* mutations appear in a particular order in the setting of drug pressure: S108 N, followed by N51I, C59R and finally I164L, with increasing resistance seen when going from two to three mutations in both *in vitro* and field isolates [78–82]. S108N appears to be a necessary first mutation in DHFR [78]. A C50R mutation was identified in samples from Latin America [72], with genetic transformation studies demonstrating that it likely has an analogous role to the C59R mutation in African isolates [83].

Mutations in *dhfr* decrease the overall enzyme efficacy and result in a fitness cost for the parasite [84, 85]. After changes in first line malaria treatment from sulfa drugs to ACTs, a decline in triple and quadruple *dhfr* mutants has been seen in certain areas [86, 87]. However, in countries where SP is part of the ACT or is used as intermittent preventive therapy, these mutants remain prevalent [88–91]. In addition, the persistence of parasites carrying *dhfr* mutations may be attributed to the use of trimethroprim-sulfamethoxazole for prophylaxis or treatment for opportunistic infections in HIV-positive persons [92].

Interestingly, *P. falciparum* parasites in Southeast Asia are able to develop a compensatory mutation for the fitness cost incurred by the mutant *dhfr*. A genome scanning study of 14 field and laboratory-adapted *P. falciparum* strains first identified an amplification surrounding GTP-cyclohydrolase 1 (*gch1*), which encodes an enzyme in the folate biosynthesis pathway that is upstream from DHFR and DHPS [93, 94] (Figure 3). A later population genetic study with a focus on the role of copy number variations (CNVs) in *P. falciparum* compared parasites from Thailand, where antifolate medications were commonly used, to those from Laos, where antifolates were rarely used [95]. They found extensive CNV surrounding *gch1* in the Thai isolates with the amplicon structure demonstrating multiple sites of origin in addition to a strong association between copy number and the *dhfr* I164L mutation, supporting how the amplification is likely an adaptive evolutionary response to antifolate therapy. The amplification reduces the cost of acquiring the drug-resistance mutations further downstream in the folate synthesis pathway [96]. A WGS study of parasites in Malawi, which experienced prolonged use of SP, found a gene duplication in a *gch1* promotor, which was also detected in parasites from West Africa and the Democratic Republic of Congo [97]. This duplication was not found as frequently in other African populations, where antifolate medications were not as extensively used and is different from the whole gene amplification found in Southeast Asia.

In *P. vivax*, the enzymes in the folate synthesis pathway are the same, and thus similar mutations in the *P. vivax dhfr* and *dhps* have been suggested to mediate resistance to the antifolate medications [98–100]. However, sulfa drugs were found to be inherently less effective against *P. vivax* parasites [101, 102]. A study that cloned and sequenced the *pvdhps* gene in multiple *P. vivax* and *P. falciparum* isolates from around the world and modeled and compared the 3-D structure of the *P. vivax* DHPS to that of *P. falciparum.* The predicted sulfadoxine-binding sites differed by one residue between the species: a valine at position 585 in *P. vivax* (probable wild type, seen in all 14 isolates examined), which is equivalent to an alanine at position 613 in *P. falciparum*. The larger valine residue of *P. vivax* at this site was predicted to reduce binding of sulfadoxine compared to the smaller alanine residue in *P. falciparum*, thus demonstrating a possible mechanism for increased inherent resistance [103]. Epidemiological studies have identified several point mutations in *dhfr* and *dhps* associated with resistance in Southeast Asia [104, 105]. A study in which recombinant, variant PvDHPS proteins were expressed showed that the mutated enzymes had reduced sulfadoxine sensitivity which correlated with higher resistance [106].

### Aryl amino-alcohols

The aryl amino-alcohols include lumefantrine (LMF) and MFQ, which are aryl amino alcohol derivatives of quinine (Table 1). Their mechanism of action is not well understood; however, they likely interfere with the detoxification of the toxic byproducts of heme degradation [107]. Quinine is a natural compound found in bark from the *Cinchona* tree that has been used for the treatment of malaria for centuries [108]. It is currently used for treatment of severe malaria and for uncomplicated malaria in the first trimester of pregnancy. LMF and MFQ were introduced shortly after the antifolate medications in the mid-1970s. However, resistance to MFQ emerged rapidly and was first reported in 1982 [109]. LMF resistance has been reported in a clinical study [110]; however this has not been confirmed [111]. LMF and MFQ are now used as partner drugs for artemisinin derivatives, and MFQ is used alone as a prophylaxis.

Resistance to MFQ was found to be primarily mediated by increased *pfmdr1* copy number [112, 113], rather than via point mutations as described for CQ and antifolate medications. Amplification of the *pfmdr1* gene has also been associated with increased risk for treatment failure with artemether-LMF [114]. The *pfmdr1* CNV is a large tandem amplification of up to 100 kb which includes several genes [115, 116]. Of note, in *P. falciparum* amplicon break points in CNVs are primarily found in monomeric tracts of A or T in intergenic regions [117]. Since the *P. falciparum* genome is highly AT-rich and has common AT monomeric tracts [118], CNVs are an important mechanism of evolutionary adaptation [117, 119, 120]. It has been found in clinical isolates throughout the world, with evidence of nonidentical chromosomal breakpoint sequences from different regions, providing evidence of independent origins [116]. This does not apply to Africa, where this amplification is rare [121]. A study of 618 samples of patients from the Thai border found that an increased copy number of *pfmdr1* was the major determinant of both *in vitro* and *in vivo* MFQ resistance [122]. The number of copies of the gene has been shown to increase the degree of resistance [123]. A study of microsatellite markers flanking *pfmdr1* and mapping of breakpoint sequences and amplicon size in clinical isolates from the Thai border found an estimated 5–15 independent origins of the amplification [117]. In contrast to the point mutations that caused CQ and antifolate resistance, which had a common origin within a population, the findings demonstrate how *pfmdr1* amplification occurs much more frequently and thus multiple independent origins can be found within a single population. The mechanism of MFQ resistance appears to be similar in *P. vivax*, with studies of clinical isolates demonstrating a correlation with *in vivo* and *in vitro* MFQ resistance and increased *pvmdr1* copy number [124, 125].

The mechanism of resistance to quinine appears to be more complex. Although there are reports of decreased sensitivity in Asia [126–128] and South America [129], high-grade resistance in the treatment of severe malaria appears to be rare [130]. *In vitro* cross resistance between quinine, the other aryl amino-alcohols and the 4-aminoquinolines is observed [123, 131–133], suggesting that there may be a common genetic mechanism of resistance. Mutations in *pfmdr1* and *pfcrt* have been found to confer decreased parasite susceptibility to quinine [18, 22, 113, 134–136]. However, they are not sufficient to cause resistance, implying that there are additional genes involved. Researchers used quantitative trait loci analysis to detect genes associated with quinine resistance in 71 *P. falciparum* isolates from diverse locations and identified *pfmdr1*, *pfcrt* and *pfnhe-1*, which encodes *P. falciparum* Na+/H+ exchanger 1 and is on chromosome 13 [137]. One of the microsatellite markers detected in *pfnhe-1* (ms4760) was significantly associated with *in vitro* response to quinine. More than two DNNND repeat motifs in block 2 of ms4760 were associated with decreased quinine response. Subsequent studies showed that an increased number of DNNND repeats were associated with *in vitro* quinine resistance [138–140]. A comprehensive analysis of *pfnhe-1* ms4760 alleles from *P. falciparum* isolates from diverse geographic locations found significant polymorphisms in these alleles, with a higher number of DNNND repeats found in Southeast Asian parasites [141].

### Artemisinin compounds

After CQ and antifolates were lost to resistance, artemisinin compounds became vital for effective malaria treatment. Artemisinin compounds are sesquiterpene lactone compounds that were discovered in China as the active ingredient in traditional medicine (extracts of the sweet wormwood plant, *Artemisia annua*) with fever-reducing properties that had been known for millennia. Related derivatives include artesunate, artemether and dihydroartemisinin (DHA) as well as the synthetic artemisinin compounds, such as OZ439. They are highly effective at rapidly clearing parasites from a person’s bloodstream. Since some have a short half-life, they typically have been combined with long-lasting drugs. These medications are currently first-line therapy as a component of ACTs. Intravenous artemisinin is used to treat severe malaria. Although their mechanism is not completely defined, within parasites these compounds undergo activation via disruption of their endoperoxide bridge, leading to oxidative stress [142] (Table 1). The precise target of artemisinin compounds is not completely defined, although current studies suggest that they cause significant stress which overpowers the parasite’s protein repair system and inactivates important housekeeping functions [142]. The phosphatidylinositol-3-kinase (PfPI3K) has been proposed as a potential target of the artemisinin compounds [143]; however the overall mechanism appears to be more complex, involving the general stress response. Treatment of *P. falciparum* with artemisinin compounds results in slowed parasite growth, decreased uptake of hemoglobin and increased oxidative damage [144]. Increased protein ubiquitination is seen in parasite following treatment with artemisinin compounds, which is likely due to substantive cellular damage [145]. One study examining the proteins covalently modified by an alkyne-tagged biotinylated artemisinin analogue identified 124 binding targets and demonstrated that heme is primarily responsible for its activation [146]. The 124 targets identified are involved in a wide variety of cellular processes and may indicate the breakdown of the general stress response rather than a specific target.

Decreased sensitivity to artemisinin compounds, as demonstrated by delayed parasite clearance (observed during clinical trials), was first reported in Cambodia in 2008 and has since emerged in other countries in the Greater Mekong region [147, 148]. One study of 91 parasites from Cambodia, Thailand and Laos used 6969 polymorphic SNPs to identify genomic regions under selection. Within these regions, analysis of SNPs and microsatellites identified a selective sweep on chromosome 13 that was associated with delayed parasite clearance following treatment with artemisinin compounds [149]. A subsequent study identified four SNPs on chromosomes 10, 13 and 14 that were associated with delayed parasite clearance time [150]. The two SNPs detected on chromosome 13 were under strong selection in the parasite population. A major breakthrough in identifying a molecular marker of artemisinin resistance was obtained in a WGS study of clinical *P. falciparum* isolates from Cambodia and a parasite line originally from Africa and selected for artemisinin resistance *in vitro*. This led to the identification of mutations in the propeller domain of the *kelch 13* gene as a mediator of artemisinin resistance [151]. The association between *kelch 13* mutations and delayed parasite clearance was subsequently confirmed with a large clinical trial [152] as well as gene editing [153, 154].

The Kelch 13 protein is thought to be involved in the cellular response to oxidative stress [142]. It is not entirely clear what specific functional changes the mutations in Kelch 13 impart; however artemisinin-resistant parasites have an enhanced stress response during the early ring stage where artemisinin is especially active [145]. Studies implicate that protein degradation or ubiquitination pathways are likely involved in this enhanced response. Transcriptional profiling of resistant parasites from patients found that proteins involved in the unfolded protein response were associated with delayed parasite clearance time [155]. The Kelch 13 C580Y variant was found to decrease interactions between the *P. falciparum* PfPI3K and artemisinin, leading to a decrease in polyubiquitination by PfPI3K and subsequent decrease in PI3P, which participates in phospholipid signaling [143]. In addition, proteasome inhibitors, which inhibit a complex that degrades unfolded proteins, were found to increase activity of artemisinin against sensitive and resistant *P. falciparum* strains [145, 156].

Epidemiologic studies demonstrate that Kelch 13 mutations have arisen independently in multiple locations in Southeast Asia, with initial soft sweeps leading to a hard sweep at this locus in parasites in Southeast Asia [157–160]. Although at least 20 mutations in K13 were identified, most parasites in the region were found to harbor a C580Y variation [151, 157]. Introduction of several *kelch 13* mutations found in field strains into isogenic parasite lines *in vitro* demonstrated different degrees of resistance, with R539T and I543T variants resulting in higher levels of resistance compared to the C580Y variant [154]. There are thus likely other factors that contribute to the widespread prevalence of a particular mutation. A GWAS study of Southeast Asian parasites showed that mutations in *pffd* (ferredoxin), *pfarps10* (apicoplast ribosomal protein S10), *pfmdr2* (multidrug resistance protein 2) and *pfcrt* were strongly associated with artemisinin resistance [159]. These mutations are thought to represent a background upon which the *kelch 13* mutations are especially likely to occur.

It is unclear what the significance of *kelch 13* mutations is in Africa where polymorphisms have been detected [161–163], but there is no clear association with artemisinin resistance. A comparison of *kelch 13* mutations between Southeast Asian and African parasites found that there was a low frequency of resistance-conferring mutations in the African parasites [164]. In addition, Asian parasites harbored an excessive number of non-synonymous mutations, while African parasites demonstrated a normal variation pattern. This suggests that these resistance-conferring mutations are not currently undergoing selection in Africa. There was one report of a returned traveler from Guinea with delayed parasite clearance with WGS showing that the strain was indigenous to Guinea and harbored a previously unreported M579I variation in Kelch 13 [165].

### Antibiotics

Antibiotics that have been used for treatment or prevention of malaria include clindamycin [166, 167] and doxycycline [168], whose mechanism of action is the interruption of protein synthesis in the parasite (Table 1). Mutations in apicoplast ribosomal RNA mediate *P. falciparum* resistance. For clindamycin, an A1875C mutation in the gene encoding the apicoplast 23S rRNA has been found in resistant field isolates that were taken into culture [169]. When tested, these parasites show resistance to clindamycin with a classic ‘delayed death’ phenotype [169]. There are no clear markers of doxycycline resistance that have been identified thus far.

### Napthoquinones

Atovaquone was developed in the 1990s and is currently used in combination with proguanil as malaria prophylaxis under the brand name malarone. Its mechanism of action is through inhibition of the electron transport chain at the cytochrome bc1 complex (Table 1). This system provides electrons for dihydroorotate dehydrogenase (DHODH), an enzyme that is responsible for *de novo* pyrimidine synthesis, which is very important for asexual blood stage parasites [170]. During clinical trials, high rates of recrudescence were seen in patients treated with atovaquone alone for *P. falciparum* malaria [171]. Resistance to atovaquone monotherapy develops rapidly and is associated with single point mutations in the gene encoding cytochrome-b [172]. Y268S/C/N mutations are found in resistant field isolates [173]. These mutations result in a significant fitness cost, since parasites harboring *cytb* mutations are unable to produce sporozoites in mosquitos rendering them untransmissible [10]. A recent study found that *P. falciparum* lines which harbor cryptic Y268S alleles in the ~22 copy mitochondrial genome can more readily evolve resistance to atovaquone *in vitro* [174]. In addition, the resistant lines demonstrated >3-fold copy number amplification of the mitochondrial genome. This suggests that the mechanism of atovaquone resistance is related to mitochondrial diversity rather than *de novo* selection of resistance mutations.

## Multidrug resistance mediators to inform ACT partner drug selection

As resistance to artemisinin emerges in Southeast Asia, there is also an increasing risk of resistance developing to the artemisinin partner drugs as parasites are effectively exposed to monotherapy. There are five partner drugs recommended by the WHO: LMF, AQ, MFQ, SP and PPQ. There are now reports of PPQ resistance emerging rapidly in Cambodia, where DHA-PPQ is a first line treatment for *P. falciparum* [58, 160, 175]. Mutations in *pfcrt* and *pfmdr1* influence *P. falciparum* sensitivity to a wide variety of antimalarial drugs, which includes quinine, MFQ, md-ADQ and artemisinin [18, 22, 23, 113, 134–136]. Clinical studies have demonstrated linkage disequilibrium between these two genes [176, 177]. The interrelatedness of mutations in these two DV membrane transporters likely reflects compensatory mutations for fitness losses or may be a mechanism to maximize drug resistance. For example, a study in which the *pfmdr1* N86Y mutation was introduced via genetic engineering into CQ-resistant and CQ-sensitive genetic backgrounds demonstrated that the mutation increased susceptibility to LMF, MFQ and DHA [41]. However, the mutation decreased susceptibility to CQ and m-ADQ in both CQ-resistant and CQ-sensitive parasites, although the decrease was more pronounced in the CQ-resistant strains. Another study that genetically edited a C101F *pfcrt* mutation into CQ-resistant *P. falciparum* found that it reversed CQ resistance and increased susceptibility to AQ, quinine and artemisinin [62]. As previously discussed, mutations in *pfcrt* are also associated with PPQ resistance. Knowledge of the mutations already present in *pfmdr1* or *pfcrt* in particular regions can inform optimal partner drug use in the setting of increasing artemisinin resistance.

A systematic analysis of the genetic changes that arose in response to 37 compounds with potent antimalarial activity detected mutations in *pfmdr1* and *pfcrt* in response to structurally diverse compounds as would be expected for pleiotropic drug transporters [178]. In addition, *pfabcI3* and *pfaat1*, genes that encode an ABC transporter and an amino acid transporter, respectively, were mutated in response to a variety of diverse compounds and likely represent multidrug resistance mediators. Interestingly, CNVs were found to contribute to one-third of the resistance acquisition events in this study.

## Parasite genetics that determine the geographic origins of resistance

The emergence of antimalarial resistance has most frequently been detected in the Greater Mekong region of Southeast Asia, as evidenced by CQ, MFQ and artemisinin resistance [4, 179, 180]. It was originally thought that parasites from this region might have a hypermutable phenotype [181], with a parasite strain from Southeast Asia demonstrating a mutation rate *in vitro* that was over 100 times greater than other clones. However, subsequent *in vitro* studies have not found a higher mutation rate in parasites derived from Southeast Asian strains [6, 182, 183]. In Southeast Asia, malaria transmission is intermittent and seasonal, which results in decreased host immunity. This may contribute to an increased propensity for drug resistance to arise. There is also a significant amount of substandard medication and poor patient compliance found in areas such as the Thai–Cambodia border that are notorious for the emergence of drug resistance [180].

## Designing new therapies

With resistance to all known antimalarial medications now apparent, the need for new medications and new approaches to treatment has become extremely urgent. In reviewing the history of antimalarial resistance and studies examining the evolution of resistance *in vitro*, there are clear lessons that can inform the design of future therapies. Resistance develops rapidly when monotherapy is employed, and thus combination therapy with at least two medications with different mechanisms of action helps mitigate this. Other important considerations to make when determining which partner drugs to use include the following: matching half-lives so that a drug with a long half-life does not persist as monotherapy, pairing drugs with synergistic mechanisms of action and avoiding a combination with antagonistic pharmacokinetics [184, 185]. Clinical trials of triple drug regimens with additional partner drugs added to established ACTs are currently underway in Southeast Asia [186, 187]. Fast acting compounds such as artemisinin are also less likely to generate resistance rapidly compared to slower-acting compounds like clindamycin or MFQ, as demonstrated by *in vitro* experiments [188, 189] and experience in the field. Drugs that have multiple cellular targets, such as artemisinin, have a higher barrier to resistance compared to drugs with a single target such as pyrimethamine.

In recent years, there has been a rapid increase in new antimalarial compounds advancing in development. Organizations such as the Medicines for Malaria Venture have partnered with academic and industrial laboratories to efficiently identify new promising antimalarial compounds for further development. There are several criteria for these compounds to fulfill, including high potency against clinical isolates from regions known for antimalarial resistance and no cross-resistance against laboratory-adapted strains that are resistant to antimalarials currently in use [190]. Another important step is determining how rapidly *in vitro* resistance occurs and what fitness cost the resistance mutations confer. Several promising compounds with new antimalarial targets have been identified, with many advancing to clinical trials [191].

The method of *in vitro* resistance evolution followed by whole genome analysis (sequencing or microarray) can identify the molecular basis of antimalarial resistance and can generate hypotheses about a new antimalarial compound’s target through comparison of SNPs acquired by the resistant clones compared to the compound sensitive parent [192]. Using this method, it was shown that resistance to Cipargamin (NITD609, KAE609), a spiroindolone drug that is the furthest along in development, is conferred by mutations in the gene encoding the plasma membrane P-type cation translocating ATPase (PfATP4) [193, 194] and this is also the likely target of the compound. Other promising compounds whose resistance mechanisms have been studied using genomic methods include the following: KAF156, an imidazolopiperazine that is active against all three parasite stages [195, 196]; DDD107498, which targets the eukaryotic elongation factor 2 [197]; bicyclic azetidines, which target phenylalanyl-tRNA synthetase [198]; imidazopyrazines, which target phosphatidylinositol 4-kinase [199], in addition to many others. Genomic analyses are also important in assessing the emergence of resistance during clinical trials for new antimalarials. DSM265 is a DHODH inhibitor that was designed using target-based drug discovery [200, 201]. During a phase 2a clinical trial, parasites from two of four recurrent *P. falciparum* infections demonstrated a resistance-associated mutation in *dhodh* [202].

## Conclusions

To review the history of antimalarial therapy is to also examine the myriad ways that the malaria parasite can develop resistance via genetic mutations. The ability to readily culture *P. falciparum* along with advances in sequencing and gene editing technologies in *P. falciparum* has greatly increased our ability to understand the effect of these mutations and confirm the changes that these mutations impart. These findings have had a direct impact on evaluating and tracking antimalarial resistance in the field, as seen most recently with the discovery of *kelch 13* mutations as a marker of artemisinin resistance. This knowledge also enables a detailed investigation into why particular treatments fail and the design of more effective antimalarial therapies.

**Summary Key Points:**

- Malaria parasites have developed resistance to all major classes of antimalarial drugs.
- Resistance to the 4-aminoquinolines and aryl-amino alcohols is primarily mediated by mutations in genes encoding transporters at the parasite’s DV membrane.
- Resistance to the antifolate drugs and atovaquone is primarily due to point mutations in the genes encoding target enzymes causing decreased binding of the drug.
- New antimalarial medications with novel drug targets are urgently needed to combat antimalarial resistance.

## Notes

**Annie N. Cowell** is an Assistant Professor of Medicine at the University of California, San Diego in the Division of Infectious Diseases. Her research interests include using whole genome sequencing of *Plasmodium* parasites from laboratory and clinical samples to better understand drug resistance and malaria epidemiology.

## Notes

**Elizabeth A. Winzeler** is a Professor of Pediatrics at the University of California, San Diego in the Division of Host Microbe Systems and Therapeutics where her group is known for (1) investigating microbial biology using genomic approaches and (2) using informatics and high throughput approaches to develop new drugs. With respect to drug development, she initiated work that resulted in two novel anti-malarial compounds that are in clinical trials (KAE609, Phase IIB and KAF156, Phase IIB) and developed high throughput assays for identifying inhibitors for exoerythrocytic stages. She is a fellow of the American Academy of Microbiology and a recipient of the ASTMH Trager award, the ASBMB Alice and CC Wang Award, the ASTMH Bailey K. Ashford Medal.
